# Identification of a new genotype of Torque Teno Mini virus

**DOI:** 10.1186/1743-422X-10-323

**Published:** 2013-10-30

**Authors:** Seyed Mohammad Jazaeri Farsani, Maarten F Jebbink, Martin Deijs, Marta Canuti, Karel A van Dort, Margreet Bakker, Bart PX Grady, Maria Prins, Formijn J van Hemert, Neeltje A Kootstra, Lia van der Hoek

**Affiliations:** 1 Laboratory of Experimental Virology, Department of Medical Microbiology, Center for Infection and Immunity Amsterdam (CINIMA), Academic Medical Center, University of Amsterdam, Amsterdam, The Netherlands; 2 Tehran University of Medical Sciences, Tehran, Iran; 3 Laboratory of Viral Immune Pathogenesis, Department of Experimental Immunology, Center for Infection and Immunity Amsterdam (CINIMA), Academic Medical Center, University of Amsterdam, Amsterdam, The Netherlands; 4 Public Health Service of Amsterdam, Cluster of Infectious Diseases, Department of Research, Amsterdam, The Netherlands

**Keywords:** Torque teno mini virus, VIDISCA-454, Virus discovery

## Abstract

**Background:**

Although human torque teno viruses (TTVs) were first discovered in 1997, still many associated aspects are not clarified yet. The viruses reveal a remarkable heterogeneity and it is possible that some genotypes are more pathogenic than others. The identification of all genotypes is essential to confirm previous pathogenicity data, and an unbiased search for novel viruses is needed to identify TTVs that might be related to disease.

**Method:**

The virus discovery technique VIDISCA-454 was used to screen serum of 55 HIV-1 positive injecting drug users, from the Amsterdam Cohort Studies, in search for novel blood-blood transmittable viruses which are undetectable via normal diagnostics or panvirus-primer PCRs.

**Results:**

A novel torque teno mini virus (TTMV) was identified in two patients and the sequence of the full genomes were determined. The virus is significantly different from the known TTMVs (< 40% amino acid identity in ORF1), yet it contains conserved characteristics that are also present in other TTMVs. The virus is chronically present in both patients, and these patients both suffered from a pneumococcal pneumonia during follow up and had extremely low B-cells counts.

**Conclusion:**

We describe a novel TTMV which we tentatively named TTMV-13. Further research is needed to address the epidemiology and pathogenicity of this novel virus.

## Background

Human torque teno virus (TTV) was first discovered in a Japanese patient with acute post-transfusion hepatitis in 1997 [1]. It is a small and non-enveloped virus, with the size of 30 nm diameter, which carries an approximately 3.8 kb, circular negative sense, single-stranded DNA genome [2,3]. According to the latest International Committee on Taxonomy of Viruses (ICTV) classification [4,5], TTV has been classified into the *Anelloviridae* family which includes also torque teno mini virus (TTMV), formerly known as TTV-like Mini virus (TLMV), and TT midi virus (TTMDV), discovered in 2000 and 2007 respectively [6,7]. The *Anelloviridae* family contains 11 genera and TTMV belongs to the genus *Betatorquevirus*, where the TTV and TTMDV are in *Alphatorquevirus* and *Gammatorquevirus* genera respectively [4,5]. All these viruses have similar genome structure but different genome sizes: 3.7-3.8 kb for TTV, 3.2 kb for TTMDV and 2.8-2.9 for TTMV [6-9].

Using alternative splicing, TTV generates three mRNA species and produces at least six proteins by alternative translation initiation [10]. TTV ORF1 is the largest ORF and encodes a capsid protein, which has an arginine-rich N terminus that is suggested to have DNA binding activity and to function in packaging of the viral DNA [3,11,12]. The noncoding region, highly conserved among TTVs [13], has a very high GC content and is around 170 bp in size. TTVs are widely spread in the whole human population and most people carry a TTV or TTMV infection but the association of TTV with disease is unclear [14-16]. Moreover, the immunological properties, the possible pathogenic role and other aspects of TTVs are still poorly understood. Persistent infection and co-infection with several genotypes are common [17-19], and some investigations suggest that TTVs are associated with hepatitis, gastritis, cancer, and acute respiratory diseases (see below), but no clear evidence has ever been presented. TTV-like sequences were identified in tumors of the gastrointestinal tract and myelomas [20]. Furthermore, several studies have mentioned the possible association of TTV with respiratory disease, asthma and its role in prognosis of idiopathic pulmonary fibrosis [21-25], while others suggested a possible association of TTV with aplastic anemia, childhood leukemia, and lymphomas [26-28]. It is also possible that the viral load, co-infections with multiple types or other agents, the immune status of the host, differences in genomic sequence or more of these factors at the same time have an influence on the pathogenicity [29]. TTVs reveal a remarkable heterogeneity and multiple genotypes have already been described [13]. It is possible that only some genotypes of TTV have a capacity to cause disease or are characterized by a higher pathogenicity than others [30]. Therefore, to have a comprehensive view on TTVs and consider their possible roles in human infections, identification of all genotypes is needed, as it represents the first step towards understanding the epidemiology, the immunology, the pathogenesis and other aspects of this virus family. In this study we describe a new genotype of TTMV identified in serum of two HIV-1 positive patients, which we tentatively named TTMV-13.

## Results

In search for novel viruses which are potentially transmitted via blood-blood contact, we screened serum of 55 HIV-1 positive injecting drug users from the Amsterdam Cohort Studies via VIDISCA-454, an unbiased virus discovery method that can detect both RNA and DNA viruses [31]. From the total of 285,400 obtained reads a novel torque teno mini virus (TTMV) was identified and its full genome was obtained via genome walking with target specific primers and nested inverse PCR.

### Genome organization and phylogenetic analysis

**Figure 1:**
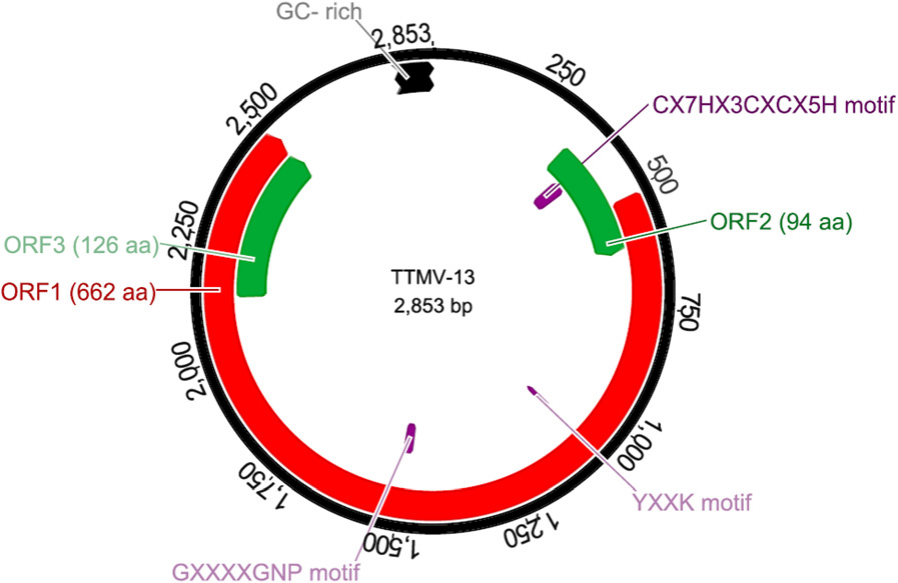
**Genomic organization of the new TTMV genotype.** Arrows represent ORFs, and the box indicates the GC-rich region. Putative proteins sizes for each ORF are given in amino acids: The ORF1 encodes a 662 amino acid long putative capsid protein, ORF2 and ORF3 a 94 and 126 amino acid putative protein, respectively. Conserved motifs in TTVs are indicated.

The genome of the virus carries the characteristic features of TTMVs (see Figure 1). The ORF1, the largest open reading frame, encodes a 662 amino acid long putative capsid protein which presents four potential glycosylation sites; no signal peptides were identified. As in other TTVs, the capsid protein is arginine rich in its N terminus and presents two conserved motives, motif 3 (YXXK) and motif 4 with 2 changes (GXXXXGNP instead of GXXXXGKS), which have been identified previously in ORF1 of other TTVs [3,32]. The second ORF encodes a putative Rep protein of 94 amino acids. ORF 2 of TTV and TTMV can contain some conserved common motifs [6,33]. The WX_7_HX_3_CXCX_5_H motif was found in the ORF2 of the new TTMV described here (CX_7_HX_3_CXCX_5_H) at the AA positions 15–34. A palindromic sequence (GGGGGCTCCGCCCCC) is repeated two times in the 3′end of genome and a GC-rich region is also present in the non-coding part, as similarly reported for TTMV, TTV and TTMDV [2,6].

A full length genome sequence was obtained from a second patient (D11, see below). In general the two viruses were very similar, with the same genome composition and overall less than 1% difference in nt identity. The variation was mainly in the ORF1 gene (1.1%), and the ORF2 genes were 100% identical at nucleotide level.

**Figure 2:**
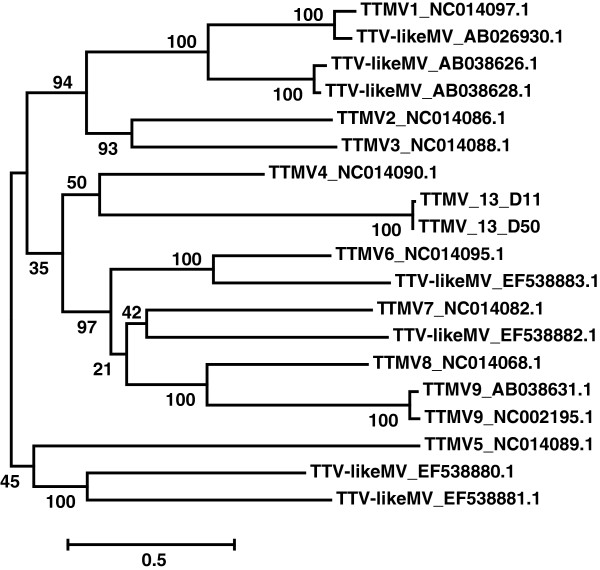
**TTMV phylogeny derived from ORF1 amino acid sequences.** ORF1 amino acid sequences (662 amino acids) of known TTMVs were aligned with TTMV-13 and phylogenetically analyzed. Numbers at nodes indicate bootstrap support (1000 replicates). The scale bar is a measure of the proportion of divergence. LogL = −20869.57.

Phylogenetic analysis shows that TTMV-13 does not cluster with any of the known TTMVs (Figure 2). The novel TTMV-13 has only 38.6% amino acid identity in ORF1 to its closest relative TTMV-4. As indicated by the scale bar in Figure 2, diversification is large among TTMVs.

### Clinical characteristics of infected patients and viral prevalence among the studied cohort

The patient (D50) in which the virus was originally detected was male, known HIV-1 positive since entry of the Amsterdam Cohort Studies, and followed for 43 months until death (April 1989 - January 1993). The patient was diagnosed with AIDS, 6 months after entering the study (pneumocystis carinii pneumonia). At the same time (6 months after study entry) zidovudine treatment was started. Seven months before death the patient developed a pneumococcal pneumonia, which was treated with sulfamethoxazole. The sample in which the TTMV was detected was collected 16 months since study entry (June 1991). Besides the novel TTMV, the VIDISCA-454 sequence reads also showed that the patient was coinfected with HCV (2,5% of the sequences) and several known members of the *Anelloviridae* (2,1% of the reads). Furthermore, HIV-1 was detected as expected (0,1% of the sequences).

To investigate whether the virus was circulating among injecting drug users, a diagnostic-TTMV13-specific-real time PCR was developed and 548 samples from 385 HIV-1 positive injecting drug users, randomly chosen from the Amsterdam Cohort Studies, were screened for presence of the virus (collection dates 1985–1995). One additional patient carrying the virus was identified. This patient (D11) was male, HIV-1 and HCV positive since entry of the Amsterdam Cohort Studies, and followed from June 1993 until death June 1998. AIDS was diagnosed 22 months since study entry (candida oesophagitis). At that time the patient had been treated with zidovudine for 20 months, which was supplemented with lamivudine after AIDS diagnosis. The patient developed a pneumococcal pneumonia and died due to a pneumococcal sepsis 5 years after study entry. The TTMV positive sample was collected 9 months since study entry (January 1994).

**Figure 3:**
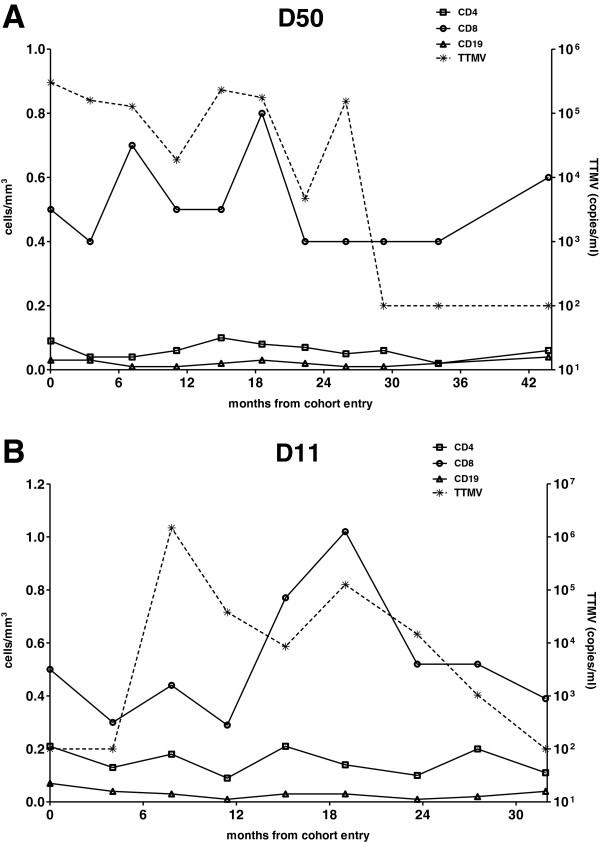
**Follow up data of patient D50 (A) and D11 (B) infected by the new genotype of TTMV.** On the X-axis the time of follow up in the Amsterdam Cohort Studies is shown, the cell counts (cells/mm^3^) on the left Y-axis and the concentration of the TTMV-13 virus in copies per ml on the right Y-axis. TTMV-13 virus loads below the detection limit (100 copies/ml serum) are indicated as 100 copies/ml.

To investigate whether the TTMV infection was chronic, sequential serum samples form both patients collected over the study period were screened for viral DNA. In both patients the virus was detectable over a prolonged period in time. Patient D50 was TTMV-13 positive during the first 26 months of follow up, whereas patient D11 became positive 7 months after study entry and cleared the virus at month 27 (see Figure 3A and B). In general, viral loads for Patient D11 were low (10^3^ -10^4^ copies/ml) except one peak in 1994.

In search for a disease association, all collected clinical and laboratory data were examined for specific factors which were in common for both TTMV-13 positive patients. Both patients have suffered a pneumococcal pneumonia during follow up and both patients had extremely low B-cells counts (both B-cells < 0.05 cells/mm^3^ blood, see Figure 3). It is possible that TTMV-13 infection is causing the low B-cell counts, suggesting that the virus is replicating in B-cells. To this end B-cells from the first patient were isolated and co-cultured with primary B-cells from a healthy donor or B cell lines (Ramos), but signs of replication – cytopathic effect, cell death or increase in viral DNA in supernatant or cells - was not observed (data not shown). A second possibility is that TTMV-13 is present at detectable levels in patients in which the immune pressure is seriously hampered due to low B-cell counts. To explore this hypothesis, we selected from the Amsterdam Cohort studies all subjects with consistently low B-cell counts (B-cells <0.05 cells/mm^3^): a total of 26 HIV-1 positive injecting drug users and 19 HIV-1 positive men having sex with men were examined for the presence of the virus but none of the serum or EDTA-plasma samples resulted positive. Attempts to express the viral capsid protein or parts of this protein in a prokaryotic protein expression system (pET100/TOPO in *E.coli* Rosetta 2 (Novagen)) were unsuccessful, therefore no serological test could be developed.

## Discussion

In this study we report a new genotype of torque teno mini virus which was discovered in serum of two HIV-1 infected patients. According to the latest ICTV update [4,5], there are 12 species of TTMV identified untill now; therefore we tentatively named the new virus TTMV-13. Based on phylogenetic inference of the amino acids sequences of ORF1, TTMV-13 is beyond doubt different from the known ones. It is known that there is a large variability among the TTVs, not only between types, but also within a certain type [34]. In that light it is surprising that the nucleotide identity between the two viruses sequenced from the two patients was remarkably high: >99% nucleotide identity. It could be that the two patients, who both shared needles, infected each other, however patient D50 deceased in 1992, whereas the first sample positive for the virus of patient D11 was obtained in 1994, therefore direct transmission of the virus is not likely.

Infection by the virus was chronic in both patients, although in both cases the virus became undetectable after a few years. It could be that the TTMV was poorly cleared by the host because of the hampered immunity, in fact both patients had low CD4 cell counts (below average) and were at the end stage of HIV-1 disease. It is known that HIV-1 affects humoral immunity through changes in the B-cell compartment [35], loss of memory B-cell subsets and B-cell exhaustion [36]. TTVs can replicate in PBMC and the viral DNA has been detected in NK cells, granulocytes cells, monocytes, B lymphocytes and T lymphocytes [23,37]. In one study TTV DNA was detected in the lymph nodes of patients with B-cell lymphomas and Hodgkin’s disease [27] and the role of TTV in lymphomas pathogenesis has been suggested [27,29]. In theory, it is possible that the low B-cell count is caused by the infection with TTMV, or a combination of an HIV-1 and TTMV infection. In one patient (patient D11, Figure 3) the low B-cell count preceded the infection with the novel TTMV-13, therefore it is not likely that the TTMV-13 was the sole cause of the low B-cell counts. Involvement of the novel TTMV-13 in pneumonia can also be considered, since it has been shown that TTMV can be detected in children hospitalized for severe pneumonia with parapneumonic empyema (PPE) and Galmes et al. suggested a possible role of TTMV in the pathogenesis of pneumonia [38]. TTVs are able to aggravate bacterial and viral infections by their ability to replicate in proliferating lymphocytes and impair the antimicrobial defenses of the host [39]. This mechanism is known for other small circular viruses such as chicken anaemia virus [40]. In some studies, association of TTV with aggravated course of multifactorial diseases such as asthma and arthritis has been suggested [24,39,41]. Furthermore, an association of TTV load with severity of acute respiratory diseases (ARD) in patients positive for community-acquired respiratory viruses (adenovirus group, cytomegalovirus, influenza A and B viruses, parainfluenza virus types 1 to 3, and RSV) has been described previously [22]. Both TTMV-13 positive patients had a pneumococcal pneumonia: one patient had pneumonia during the period in which TTMV-13 was detectable in blood and the second patient developed pneumonia when TTMV-13 was no longer detectable in blood (patient D11). It still could be that the virus had continued replicating at a lower level in patient D11, with viral loads below the detection limits of our diagnostic assay. In general, the concentration of the virus in blood was constantly low, with exception of a viral load peak in 1994 for what regards patient D11, which might have been the time of primary infection. Later time-points were negative or close to the detection level of our diagnostic PCR assay thus prolonged and continued infection with low levels of virus in blood could have been missed.

## Conclusion

With the discovery of the novel TTMV-13 we are one step further with revealing the full spectrum of genotypes and sequence variation among anelloviruses.

## Methods

### Clinical samples

Serum samples of HIV-1 infected individuals were obtained from the Amsterdam Cohort Studies on HIV infection and AIDS [42]. The selected patients were injecting drug users who were HIV-1 positive at entry of the Amsterdam Cohort Studies. Samples (N = 55) for VIDISCA-454 (virus discovery cDNA-AFLP, Amplified Fragment–Length Polymorphism combined with Roche 454 high-throughput sequencing) were collected at least 2 years since study entry and were selected to be from patients whose CD4 counts decreased below 0.3 cells/mm^3^ (characteristics are shown in Additional file 1: Table S1). The Amsterdam Cohort Studies has been conducted in accordance with the ethical principles set out in the declaration of Helsinki and written informed consent has been obtained. The study was approved by the Academic Medical Center institutional medical ethics committee.

### VIDISCA-454 and full-length genome sequencing and characterization

VIDISCA-454 was performed with 110 μl of serum/EDTA-plasma samples as previously described [31]. Full length sequencing of the virus was performed via genome walking starting from fragments identified with VIDISCA-454. After extraction of nucleic acids from the serum samples [43], DNA walking (Seegene) with target specific primers was used together with a nested inverse PCR. Primers used for sequencing are shown in Additional file 2: Table S2. PCR products were cloned into pCR2-TOPO vector and sequencing was performed employing Big Dye terminator chemistry (BigDye® Terminator v1.1 Cycle Sequencing Kit, Applied Biosystems). Sequences were analyzed with Codoncode Aligner software (version 3.7.1). Open reading frames were identified via ORF finder (http://www.ncbi.nlm.nih.gov/gorf/gorf.html). N- and O-linked glycosylation sites and signal peptide cleavage sites were predicted using the NetNGly 1.0, NetOGly 3.1, and SignalP 4.0 analysis tools, from the Center for Biological Sequence Analysis (http://www.cbs.dtu.dk/services/).

### Real time PCR

Nucleic acids were extracted from serum samples using the Boom method [43]. Absolute quantification of viral DNA (copies/ml of serum) was achieved by TTMV-13-specific real-time qPCR kit (Platinum® Quantitative PCR SuperMix, invitrogen) using the forward primer, 5′-ACAACACACATGGGAAAATGCA-3′, reverse primer, 5′-CTTTTGGTGTGTTGCCCTGTTG-3′, and the probe, 5′-FAM-TATTTTTAGGAAACACATTAGACAA-TAMRA-3′. Real-Time PCR was performed on an ABI Prism 7000 Sequence Detection System machine. A positive control was prepared by cloning PCR products into the pCR2-TOPO plasmid vector according to the instructions of the manufacturer (Invitrogen, Karlsruhe, Germany) followed by plasmid DNA isolation with QIAprep Spin Miniprep Kit (Qiagen, Hilden, Germany). Plasmid concentration was quantified using Nanodrop 2000 (Thermo Scientific) and a standard curve with 1:10 serial dilutions was prepared as reference for DNA quantification. The detection limit for the assay was 100 copies/ml.

### Phylogenetic analysis

Available complete TTMV genome sequences in GenBank database (http://www.ncbi.nlm.nih.gov/genbank/) were used for the analysis (accession numbers are listed in Figure 2). Redundancies were removed and the few nucleotide ambiguities were solved via replacement by either the consensus nucleotide at the position involved or the corresponding nucleotide of the nearest neighbor. At absence of these opportunities, a gap was introduced in the translated reading frames. The amino acid sequences of ORF1 were aligned by means of ProbCons [44] using default parameters. Phylogenetic and molecular evolutionary analyses were conducted using MEGA version 5.2 [45]. The WAG + Freq + G + I model for assessing amino acid replacements during TTMV evolution turned out to be the best fitting model judged by BIC score (Bayesian Information Criterion, 42296.3052) and AICc value (corrected Akaike Information Criterion, 41880.2816) and therefore used for the following Maximum likelihood phylogenetic analysis via Mega5 [45]. Non-uniformity of evolutionary rates among sites has been modeled by using a discrete Gamma distribution (+G = 2.3418) with 5 rate categories and by assuming that a certain fraction of sites is evolutionarily invariable (+I = 0.0280). Maximum Likelihood (ML) value for model selection was logL = −20883.8854. In view of the relatively low number of taxa, all sites were used for phylogenetic analysis. To test the robustness of the analysis, a bootstrap test (1000 replicates) was performed and only clusters associated with a value higher then 75% were considered significant [46].

### Accession numbers

Two complete genome sequences of TTMV-13 obtained in this study have been deposited in the GenBank database under accession numbers KF764701 and KF764702.

## Abbreviations

TTV: Torque teno virus; TTMV: Torque teno mini virus; TLMV: TTV-like mini virus; VIDISCA: Virus discovery cDNA-AFLP; ICTV: International committee on taxonomy of viruses; HIV: Human immunodeficiency virus; AIDS: Acquired immunodeficiency syndrome; ARD: Acute respiratory disease; HCV: Hepatitis C virus; RSV: Respiratory syncytial virus; PPE: Parapneumonic empyema; PBMC: Peripheral blood mononuclear cell; ORF: Open Reading Frame.

## Competing interests

The authors declare they have no competing interests.

## Authors’ contributions

SMJF carried out the characterization of the virus infections, the prevalence of the infection, the full length sequencing and the writing of the manuscript. MFJ participated in protein expression, MD, MC and SMJF performed the VIDISCA-454 and analyzed the sequence data, KAD and NK carried out the virus culture, MB analyzed the clinical, cell biology and virology data of the patients, BPXG, MP collected the clinical and behavioral data. MC and FJH performed the phylogenetic analysis. LH designed the study and participated in drafting the manuscript. All authors read and approved the final version of the manuscript.

## Supplementary Material

Additional file 1: Table S1 HIV-1 Virus load and CD4 cell counts of the VIDISCA-454 analysed patients. Click here for file

Additional file 2: Table S2 Primers used for full length genome sequencing. Click here for file
