# Supplementary material for: Identification of a new genotype of Torque Teno Mini virus
Source: Virol J. 2013 Oct 30;10:323. doi: 10.1186/1743-422X-10-323 (PMC3819664; doi:10.1186/1743-422X-10-323)
Supplement: Additional file 1: Table S1 — HIV-1 Virus load and CD4 cell counts of the VIDISCA-454 analysed patients. [file 1743-422X-10-323-S1.doc]

**Additional file 1: Table S1. HIV-1 Virus load and CD4 cell counts of the VIDISCA-454 analysed patients**

| **code sample** | **Gender** | **CD4 cell counts, cells/ mm^3^** | **HIV-1 virus load, RNA copies/ml serum** |
| --- | --- | --- | --- |
| 2010-20 01 | V | 0,29 | 14660 |
| 2010-20 02 | V | 0,19 | 38875 |
| 2010-20 03 | V | 0,25 | 63080 |
| 2010-20 04 | V | 0,29 | 1465 |
| 2010-20 05 | V | 0,09 | 1955 |
| 2010-20 06 | V | 0,23 | 34630 |
| 2010-20 07 | M | 0,29 | 11465 |
| 2010-20 08 | V | 0,27 | 9165 |
| 2010-20 09 | V | 0,18 | 67805 |
| 2010-20 10 | V | 0,25 | 16100 |
| 2010-20 11 | V | 0,12 | 28495 |
| 2010-20 12 | V | 0,19 | 33165 |
| 2010-20 13 | V | 0,07 | 109235 |
| 2010-20 14 | V | 0,18 | 36430 |
| 2010-20 15 | M | 0,22 | 27485 |
| 2010-20 16 | M | 0,25 | 63540 |
| 2010-20 17 | M | 0,23 | 22130 |
| 2010-20 18 | M | 0,26 | 780 |
| 2010-20 19 | V | 0,22 | 480 |
| 2010-20 20 | M | 0,05 | 50790 |
| 2010-20 21 | V | 0,19 | 30630 |
| 2010-20 22 | V | 0,12 | 34385 |
| 2010-20 23 | M | 0,15 | 34385 |
| 2010-20 24 | M | 0,21 | 18205 |
| 2010-20 25 | V | 0,28 | 7875 |
| 2010-20 26 | M | 0,18 | 4385 |
| 2010-20 27 | M | 0,02 | 114075 |
| 2010-20 28 | M | 0,18 | 412755 |
| 2010-20 29 | V | 0,12 | 5295 |
| 2010-20 30 | M | 0,18 | 375 |
| 2010-20 31 | M | 0,29 | 6575 |
| 2010-20 32 | M | 0,21 | 72890 |
| 2010-20 33 | M | 0,18 | 4855 |
| 2010-20 34 | M | 0,14 | 256215 |
| 2010-20 35 | M | 0,07 | 86065 |
| 2010-20 36 | M | 0,22 | 1011020 |
| 2010-20 37 | V | 0,01 | 5490 |
| 2010-20 38 | M | 0,17 | 6915 |
| 2010-20 39 | M | 0,26 | 15310 |
| 2010-20 40 | V | 0,23 | 189160 |
| 2010-20 41 | M | 0,29 | 150 |
| 2010-20 42 | M | 0,22 | 975 |
| **2010-20 43 (D50)** | **M** | **0,05** | **26700** |
| 2010-20 44 | M | 0,25 | 2785 |
| 2010-20 45 | M | 0,29 | 7650 |
| 2010-20 46 | M | 0,18 | 4995 |
| 2010-20 47 | M | 0,2 | 2160 |
| 2010-20 48 | M | 0,08 | 5770 |
| 2010-20 49 | M | 0,24 | 11975 |
| 2010-20 51 | V | 0,29 | 11140 |
| 2010-20 52 | M | 0,17 | 16220 |
| 2010-20 53 | M | 0,26 | 16575 |
| 2010-20 54 | V | 0,05 | 11975 |
| 2010-20 55 | M | 0,14 | 8345 |
| 2010-20 56 | M | 0,25 | 24840 |
