# Supplementary material for: Identification of a new genotype of Torque Teno Mini virus
Source: Virol J. 2013 Oct 30;10:323. doi: 10.1186/1743-422X-10-323 (PMC3819664; doi:10.1186/1743-422X-10-323)
Supplement: Additional file 2: Table S2 — Primers used for full length genome sequencing. [file 1743-422X-10-323-S2.doc]

**Additional file 2: Table S2. Primers used for full length sequencing of TTMV13**

TTMV1_R: TTAAAAATAATGCACAACACACA

TTMV1_F: CATTAGACAACCAAGAGGG

TTMV2_F: CCTGCTCCTTAAATGAATGG

TTMV2_R: CCATTCATTTAAGGAGCAGG

TTMV3_F: GACTTAAAGAATATCAACCAAG

TTMV3_R: CTTGGTTGATATTCTTTAAGTC

TTMV4_F: CAACCTCACTATAATTTACC

TTMV4_R: GGTAAATTATAGTGAGGTTG

TTMV5_F: CTAAGTACACTGACGAATGG

TTMV5_R: CCATTCGTCAGTGTACTTAG

TTMV6_F: GTGTCTGTTCCTTTAAGTCTC

TTMV6_R: GAGACTTAAAGGAACAGACAC

TTMV7_F: GAGGATAAACTTTTGTTATAG

TTMV7_R: CTATAACAAAAGTTTATCCTC

TTMV8_F: CTTCTTCTTCCTCGGATGAAC

TTMV8_R: GTTCATCCGAGGAAGAAGAAG
